# The Implications of the COVID-19 Pandemic on the Interest in Orthodontic Treatment and Perspectives for the Future. Real-Time Surveillance Using Google Trends

**DOI:** 10.3390/ijerph18115647

**Published:** 2021-05-25

**Authors:** Magdalena Sycinska-Dziarnowska, Hanna Bielawska-Victorini, Agata Budzyńska, Krzysztof Woźniak

**Affiliations:** Department of Orthodontics, Pomeranian Medical University in Szczecin, Powstańców Wielkopolskich Street 72, 70111 Szczecin, Poland; hania.bielawska@wp.pl (H.B.-V.); klimaga@op.pl (A.B.); krzysztof.wozniak@pum.edu.pl (K.W.)

**Keywords:** orthodontic treatment, invisalign, COVID-19 pandemic, Google Trends

## Abstract

Background: The COVID-19 pandemic outbreak may have a large impact on orthodontic treatment. Regular orthodontic visits were strongly and widely interrupted by the unprecedented epidemiological threat. Methods: The data regarding orthodontic queries were investigated in a real-time surveillance using Google Trends. Search terms “braces”, “invisalign”, “get braces”, “get braces off”, “braces pain” and the phrase “social distancing” were analyzed regarding the year preceding the pandemic outbreak and the time of the pandemic. Moreover, the five-year trend for queries “braces” vs. “invisalign”, as an example of different types of orthodontic appliances, was compared. Results: There was a significant decrease in orthodontics phrase queries in the spring of 2020, connected with the worldwide restrictions and lockdowns announced all over the world. There was a lower interest in the “braces pain” query during the first lockdown in 2020. The number of searches for “invisalign” increased steadily over time, while the number of searches for “braces” was relatively stable across the investigated time period. Conclusions: The course of the COVID-19 pandemic has had a large impact on the orthodontic-related search queries. Orthodontists must be better-prepared for any sudden changes in the possible future in the epidemiological situation that may change accessibility to dental offices.

## 1. Introduction

The unprecedented nature of the COVID-19 pandemic outbreak has shown how health aspects are important for the whole population [1]. During the spring lockdown in 2020 and because of social distancing, only essential services were left open. Oral dental professionals were significantly affected by the COVID-19 pandemic, due to the mass closure of dental offices [2,3]. The pandemic has had a continuing effect on the orthodontic treatment, as the orthodontic treatment is a long process that requires regular visits [4]. Thus, thousands of orthodontic patients already undergoing treatment missed their monthly visits [5]. According to some authors, regular orthodontic visits should be suspended during the COVID-19 pandemic due to the high risk of virus transmissions. If they were to be carried out, dedicated screening of the patients as well as triage, stringent infection control protocols and higher ventilation and disinfection should be introduced in orthodontics offices. The federal, state or local public health guidelines should be followed as well during the pandemic [2,5,6]. According to Bilder et al., 85% of orthodontic patients experience an emergency visit during the orthodontic treatment [7]. An orthodontic emergency may occur or escalate in a very short period and need urgent intervention. In addition, according to American Association of Orthodontists, emergencies may lead to oral mucosa or gingiva infection or severe pain [8,9]. Moreover, the prolonged time without orthodontic visits may increase the number of patients with mental stress and anxiety [10]. It was proved that 31.1% of orthodontic patients undergoing treatment were concerned about the treatment being prolonged [11].

First of all, the dynamically changing everyday situation during the pandemic has to be better studied in order to keep the quality of life at an optimal level [2], as well as to provide in the near future a wide spectrum of preventive actions to promote oral health and regular dental and orthodontic visits. Moreover, the data acquired via the Internet search engines give quick and up to date information about new challenges regarding oral health. Before the pandemic outbreak other orthodontic-related subjects were discussed on social media [12,13]. Additionally, in the year 2014, scientists proved that the Internet became for some patients an alternative way to look for medical information as well as gaining first-hand information from people who had experienced the same situation [14]. Google Trends (GT) can detect the world-wide community attitude and trend, not only personal ones, and since the data are publicly available, it may help to investigate the oral health needs during unforeseen situations and when there is a lack of regular dental office visits [15,16].

In view of the insufficient research on the subject, the aim of this study was to investigate the influence of the COVID-19 pandemic on orthodontic treatment based on the Internet search queries performed in the Google search engine.

## 2. Materials and Methods

The GT tool was used to collect data on the Internet search interest among anonymous Google search engine users related to the orthodontic topics. In this free and publicly available service, data is available according to the chosen time period [15,16]. All data collected for the study is publicly available in the GT service. The data is normalized, ranging from 0 to 100, where 100 means the maximum number of searches in a stated period of time. In the study, the examined material was gathered from the 1 January 2019 to the 28 February 2021 and the values were collected according to weekly intervals. Search terms were retrieved globally and stated in the English language.

The orthodontic phrases “braces”, “invisalign”, “get braces”, “get braces off”, and “braces pain” were analyzed and the phrase “social distancing” was checked and compared to indicate the period of the first lockdown due to the pandemic situation in 2020. Moreover, in the period of the last five years starting from March 2016, the results for phrases “braces” and “invisalign” were collected in order to compare the trend between different types of orthodontic appliances.

The statistical analysis was performed according to seven time periods. The intervals were chosen according to the approximate pandemic phases in the year 2020 rounded up to months. In 2020, there were three periods chosen: the first related to the pre-pandemic phase, which was set for the months January and February; the second from the beginning of March till the end of June, at the initial phase of the pandemic; and the third starting from the beginning of July until the end of December. To compare the results with the previous year and the beginning of the year 2021, the corresponding periods were investigated (January to February, March to June, July to December 2019 and January to February 2021). This division was conducted to find out if a statistically significant difference between expressions in the aforementioned subperiods appeared.

The R 4.0.2 software was used for the statistical analyses. The level of statistical significance was set at α = 0.05. To test for the relationship of the standardized number of searches between the pairs of phrases within selected time periods, Pearson correlation coefficient was used. In one case, a nonlinear relationship was noted; thus, linear regression with a second-degree polynomial was used to model the association between the variables. For a five-year time series of a standardized number of searches for the phrases “braces” and “invisalign”, a rolling average with sixteen periods (weeks) was used to present the trend.

The daily GT normalized search volume was transferred to the spreadsheet and visualized on the charts. Relative search volume (RSV) charts were used to show changes in time for the investigated phrases.

## 3. Results

**Figure 1:**
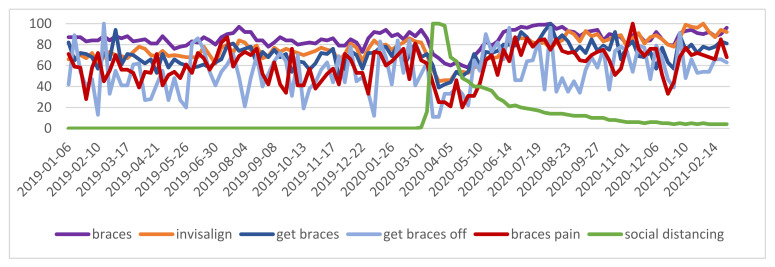
Worldwide phrases—relative search volume.

The decrease in orthodontics phrases in the year 2020 was in line with the worldwide restrictions announced by governments [1]. The peak of the “social distancing” expression was observed in March 2020, probably due to the coronavirus orders given worldwide by the governments [17] (Figure 1). In May, during the weeks of the lowest number of queries for orthodontic phrases, a local peak for the phrases “braces pain” and “get braces off” was observed; however, it was not statistically significant (Figure 1).

**Figure 2:**
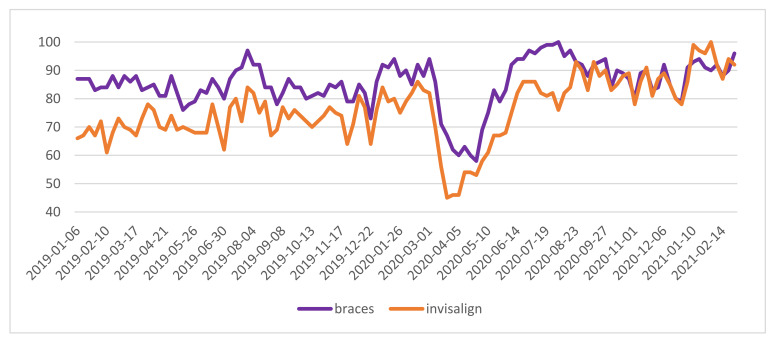
“braces” and “invisalign” phrases—relative search volume.

The “invisalign” trend imitated the “braces” trend, with an even slightly stronger decrease of interest in the “invisalign” search, during the spring lockdown and larger increase in searches for “invisalign” at the beginning of 2021 (Figure 2). The lowest values of GT relative search volume (RSV) for “braces” and “invisalign” were observed in March and April of 2020 (Figure 2).

**Figure 3:**
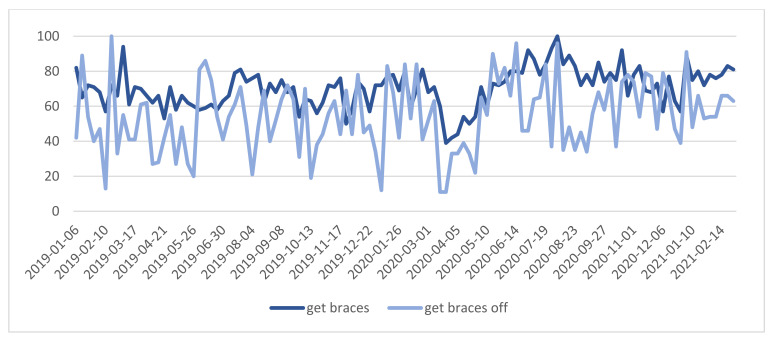
“get braces” and “get braces off” phrases—relative search volume.

On the contrary, the highest RSV for “braces” and “get braces” was observed at the beginning of August 2020 (Figure 2 and Figure 3).

**Figure 4:**
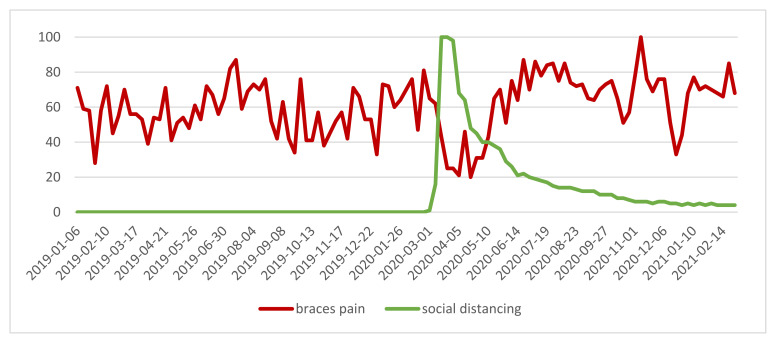
“braces pain” and “social distancing” phrases—relative search volume.

The highest number of questions regarding “braces pain” were asked at the beginning of November 2020 (Figure 4).

In the performed correlation analyses between the standardized number of searches for “braces” and “invisalign” phrases, it was observed that the number of searches for these phrases were significantly, positively and strongly associated during the time period 03/2020 to 06/2020, with r = 0.71 and *p* < 0.001. In the 07/2020 to 12/2020 period, the relationship was also strong but nonlinear with r-sq. = 0.7 and *p* < 0.001. There was similar behavior of interest in “braces” and “invisalign” expressions during the first lockdown in the spring of 2020. However, the situation was much different in the second half of 2020, where the nonlinear relationship showed both a positive and negative relationship; rare queries for “braces” were related to rare and frequent queries for “invisalign”, and when the average number of queries for “braces” were observed, there were the most frequent “invisalign” phrase search queries.

In the correlation analyses between the standardized number of searches for “get braces” and “get braces off” phrases, it was observed that the number of searches for these phrases were significantly, positively, and strongly associated during two time periods: 03/2020 to 06/2020 with r = 0.68, *p* = 0.002 and 01/2021 to 02/2021 with r = 0.91, *p* = 0.001. This indicates that during the first spring lockdown in 2020, the interest in the “get braces” phrase as well as in the “get braces off” expression behaved similarly and the same situation was observed at the beginning of 2021. Neither the prevalence in getting new orthodontic appliance nor the desire to finish orthodontic treatment was statistically proven.

The number of searches for “social distancing” was zero until 02/2020 due to no interest in this phrase. The correlation analyses between the standardized number of searches for “braces pain” and “social distancing” phrases showed a strong negative relationship between the phrases in the 03/2020 to 06/2020 period with r = −0.69 and *p* = 0.002. It shows that the more interest there was in the “social distancing” expression, the lower the interest in the “braces pain” phrase occurred. The increase in the number of queries for “braces pain” occurred in the later phase of the pandemic—07/2020 to 12/2020—and there was a moderate positive statistical relationship, with r = 0.48 and *p* = 0.013.

According to other investigated time periods, statistically significant correlations were not observed.

**Figure 5:**
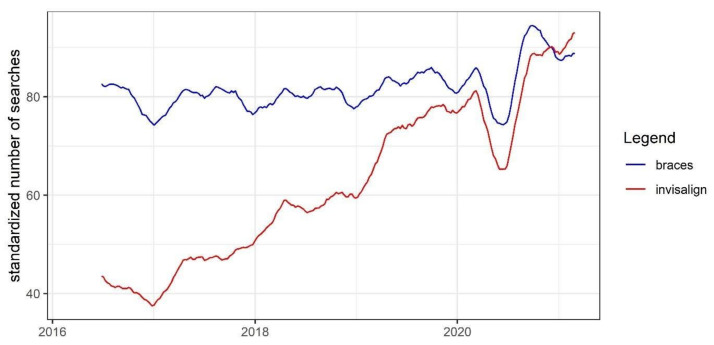
Smoothed standardized weekly number of searches for “braces” and “invisalign”.

Smoothed five-year trends of the standardized number of searches for “braces” and “invisalign” phrases are presented in Figure 5. The number of searches for “braces” was relatively stable across the selected time period, while the number of searches for “invisalign” increased steadily over time. However, there was a notable decrease in the number of searches for both phrases observed in the spring of the year 2020.

## 4. Discussion

The aim of the study was to investigate the impact of the COVID-19 pandemic on orthodontic subjects. To the best of our knowledge, this is the first study tracing the orthodontics expressions in the Google search engine. The first noticeable value of the research is a large decrease in all orthodontics questions asked during the spring lockdown in 2020. It was statistically proven that no such behavior was observed in the year preceding the COVID-19 pandemic outbreak. The beginning of the lockdown was indicated in the study by the “social distancing” phrase that, before the stringent control measurements, did not occur in the Google queries.

Quite unexpectedly, in the present study, there was no substantial increase observed in the interest according to the phrase “braces pain” during the first lockdown period that occurred in the spring of 2020. A lot of orthodontics offices were closed due to the COVID-19 disease [18], which caused the cancellation of orthodontic visits, especially in the early phase of pandemic outbreak [6] as it was recommended to consider only urgent cases [2]. The same measure to control the viral spread was undertaken in the year 2003; dental visits were postponed for at least a one-month period for patients with SARS [19]. However, the two epidemic outbreaks are difficult to compare because of the larger scale of the ongoing pandemic. According to the study conducted in Brazil in May 2020; 66.8% of 395 orthodontist who answered an online survey were handling only emergencies and 14.2% closed their offices during COVID-19 pandemic. On the other hand, 19% of orthodontist were providing routine orthodontic visits [20].

The Invisalign aligner is an optional orthodontic appliance that does not cause pain due to protruding wires or other unexpected orthodontic harmful failures, as opposed to fixed orthodontic appliances. In the study of Cotrin et al., 74.7% of emergences were related to stainless steel appliance compared to aligners, which were mentioned by 5.1% of orthodontists. Additionally, less emergences with aligners breakage were observed at 8.7% vs. bracket damage in 67.6% of emergency cases with the breakage of the archwire that caused mucosal injury [20]. This could have led to the larger amount in “invisalign” queries in later months of the pandemic. In contrast, the multibracket straight wire appliance needed more direct interventions of the orthodontist due to the more complicated oral hygiene maintenance and the need to solve emergency problems [21]. This is in line with the study conducted by Jones et al. [22] as the most common emergency in orthodontic studio was caused by debonded brackets (38%). Similarly, the most frequent emergency appointment was due to a loose bracket or buccal tube [23]. In contrast, the most common problem with orthodontic appliance was a protruding wire, problematic in 30%, but closely followed by a debonded bracket, reported by 27.3% of the respondents [24]. However, in our research, no significant statistical difference was observed in the interest between the queries “invisalign” and “braces” during the 2020 spring lockdown. On the other hand, as the year 2020 has shown the new advantages or disadvantages of different type of treatment, the increase in the “invisalign” queries was observed when compared to the five-year period. The growing interest in the “invisalign” method during the ongoing months of the COVID-19 pandemic was observed. Another advantage for the patients treated with aligners is that they can reuse the previous aligner or use the subsequent aligner when the current one is broken or lost, and it often does not need a visit to the dental office [25]. According to previous studies, the aligners had significant advantages when comparing the treatment duration and chair time [26,27]. Furthermore, no differences in stability after treatment was found between the two systems [26], on the other hand, Kuncio et al. [28] proved that aligner cases relapsed more than the fixed appliance cases. When compared with fixed appliances, the clear aligners have better esthetic and oral hygiene and aligners are less affected by orthodontic emergencies and skipped visits if the patient is provided with a set of aligners [10,27,29]. In line with the conducted study, more interest in the “invisalign” phrase was observed in the second half of the year 2020.

However, good compliance is needed as clear aligners are much more patient-dependent when compared to fixed braces and the patients’ motivation to undergo the treatment must be high; otherwise, it is impossible to gain the desired results [27]. The problem with cooperation with the patient may arise due to the fact that one-third of orthodontic patients experienced mental stress during the ongoing pandemic (38%). The manner of communication between patient and doctor may reduce patient anxiety, especially when the patient was informed of the suspended appointments from the doctor, not from the Internet [10]. The main reasons for anxiety were orthodontic treatment duration and the final treatment outcome. On the other hand, only 44.2% of patients under treatment were to visit their dentist only in an emergency case, while 17.5% would refuse to visit in any case [11]. In our study, this thesis may be supported by the large decrease during spring lockdown in queries for “braces pain” or “get braces off”. Previous literature based on the SARS outbreak in Hong Kong in 2003 has suggested that even healthcare students were affected by fear of the new disease [30]. In the more recent study, a higher level of mental distress and anxiety was observed among orthodontics patients (48.7%) when compared to patients undergoing other dental procedures (27.4%) [11]. Gao et al. [31] proved that patients treated with fixed appliances experienced a higher level of pain and anxiety than the group receiving aligners. The problems with the longer lack of treatment occurred in the later phase of the pandemic—07/2020 to 12/2020—with the peak for the “braces pain” phrase in November 2020 when compared to the lower interest in that phrase during the lockdown.

The most frequently asked question by orthodontic patients is: how long will I have to wear braces? Many factors determine this answer, which has significant importance for patients. The delay of the orthodontic visits for more than two months led to the need of emergency appointments or reassurance and consultation [24]. According to Xiong et al. [10], 90.39% of orthodontic patients had their orthodontic appointment delayed for over one month. The authors of the above-mentioned study learned that over half of the patients rarely communicated with their orthodontist due to the dental clinics being closed. Beckwith et al. [32] reported that the orthodontic treatment was prolonged for 1.09 months with each missed appointment. This is in line with our outcomes, with less interest in orthodontics search queries. In the conducted study, a large decrease in the quantity of orthodontic search queries was noted after the pandemic outbreak.

Previous literature suggested that tele-orthodontics reduces the time spent in the dental office [21]. Moreover, many of the orthodontics emergencies could have been solved by tele-orthodontics without urgent need of a dental chair visit [33]. The literature demonstrated that aligners—for example, Invisalign—needed only a follow-up to continue treatment, which can be provided via tele-orthodontics [21]. According to previous research, tele-orthodontics could help in orthodontic emergencies, as well as facilitate communication with the patient and provide virtual counseling [21,34]. It might also make it easier to resolve emergency orthodontic problems and help to decide whether the patient can solve the problem at home or needs an emergency visit. The authors advise to first try and find a solution to the problem remotely [5]; the online triage for the post-COVID-19 patients could also facilitate work at dental offices [35]. A useful solution might be, for example, WhatsApp messenger, used by 83.8% patients in the Brazilian study [20].

The limitation of the study is the use of only one search engine. However, the Google engine was chosen as most widely used, with more than 92% of the worldwide search engine market share [36,37]. Another limitation occurs when it comes to the selection of investigated expressions; due to the insufficient quantity of queries for “aligners”, the trademark “invisalign” was examined, as it met the study requirements.

## 5. Conclusions

1. The pandemic outbreak, especially in its early phase, greatly influenced and changed people’s interest regarding the orthodontic-related queries asked in the Google engine.
2. Much fewer queries were asked regarding “braces pain” during the first 2020 lockdown, which may demonstrate a widespread fear of the unknown virus.
3. The five-year observation showed growing interest in the “invisalign” trend, which was even more evident in 2021, after the experience of lockdown that brought fears and changes to many aspects of life.
4. Orthodontists must be aware of new threats and become better prepared for future changes in the epidemiological situation that may rapidly change accessibility to dental offices.

## Data Availability

The data presented in this study are available on request from the corresponding author.
